# University students’ cognitive flexibility and critical thinking dispositions

**DOI:** 10.3389/fpsyg.2024.1420272

**Published:** 2024-09-09

**Authors:** İsmail Karakuş

**Affiliations:** Department of Basic Education, Faculty of Education, Mersin University, Mersin, Türkiye

**Keywords:** cognitive flexibility, critical thinking, critical thinking disposition, relational survey model, university students

## Abstract

The purpose of this study is to examine whether there are differences in critical thinking dispositions and cognitive flexibility among university students based on gender, grade level, and faculty. Additionally, the study will investigate the relationship between these two concepts and their predictive power. The study was conducted using a relational survey model and included 366 university students selected through maximum diversity sampling. The study involved university students from various faculties and grade levels. Data was collected through a personal information form, cognitive flexibility inventory, and critical thinking disposition scale. The data was analyzed using the SPSS 25 program. The results indicate that university students exhibit relatively high levels of cognitive flexibility and critical thinking tendencies. Above the medium level, there was a significant positive relationship between cognitive flexibility and critical thinking tendency. Cognitive flexibility was found to be a significant predictor of critical thinking dispositions, positively and significantly predicting critical thinking disposition and explaining 40% of it. Individuals with critical thinking tendencies exhibit cognitive flexibility, which is also associated with thinking critically. Therefore, cognitive flexibility and critical thinking are interrelated characteristics.

## Introduction

In daily life and learning processes, individuals must be able to adapt to new situations, find solutions to problems, and cope with new challenges. Cognitive flexibility, as explained by Bonnici (2020), is defined as an individual’s ability to adapt to new or unexpected situations, to switch between various tasks and to evaluate different perspectives. Cognitive flexibility refers to the capacity to adjust to new situations, creatively solve problems, recognize available options, willingly apply them, evaluate using regulatory strategies, and feel competent in these matters (Kloo et al., 2010; Buğa et al., 2018). Cognitive flexibility is a crucial trait that influences an individual’s capacity to adjust to dynamic environments and goal-oriented behaviors (Gabrys et al., 2018).

Cognitive flexibility is a concept that refers to an individual’s ability to perceive difficult situations as controllable, generate alternative solutions, and perceive multiple alternatives for life events and human behavior. Similarly, Dennis and Vander Wal (2010) described it as the tendency to perceive difficult situations as controllable, the ability to perceive multiple alternatives for life events and human behavior, and the ability to generate alternative solutions. It is important to note that cognitive flexibility is a crucial skill for managing pressure in difficult and complex situations. This skill allows individuals to adapt to situations by developing different perspectives, ultimately leading to problem-solving (Diamond, 2021).

Cognitive flexibility comprises three fundamental elements: awareness of different solutions and options in stressful situations, willingness to adapt to flexible situations, and self-efficacy (Wolff et al., 2017; Asıcı and İkiz, 2015). It is closely related to problem-solving skills (Bedel and Ulubey, 2015), communication skills (Smith and Davis, 2022), and adaptability (Dunleavy and Martin, 2006; Peker and Çukadar, 2016). Individuals with cognitive flexibility are expected to cope effectively with new and difficult situations, produce alternative thoughts and ideas, and adapt to new situations (Arslan and Türk, 2022; Altunkol, 2011). Meanwhile, studies have suggested that individuals who engage in this behavior exhibit increased self-confidence, attentiveness, and understanding (Çelikkaleli, 2014). They are also known to possess strong communication skills, sociability, and a sense of responsibility (Ghazanfari and Pourhosein Gilakjani, 2022; Lan, 2022; Rezaei and Jafari, 2023). Additionally, they are capable of replacing negative thoughts with more positive ones, developing new coping mechanisms, and perceiving challenging situations as more manageable (Arslan and Türk, 2022). In addition to these abilities, students are expected to possess critical thinking skills (Yeşilyurt, 2021; Tümkaya and Aybek, 2008). Critical thinking skills are based on cognitive abilities (Gök and Erdoğan, 2011).

Thinking is considered a fundamental skill and phenomenon that distinguishes humans from other living beings and influences our lives (Yeşilyurt, 2021). Critical thinking begins when individuals start to explore events that impact their behavior, whether they are natural or social (Brookfield, 2012). Critical thinking requires research, logical inferences, questioning, and organizational skills (Eales-Reynolds et al., 2013). These skills are essential for finding solutions to problems in education, business, and social settings, as well as for evaluating the reliability of information and making informed decisions.

There are numerous definitions of critical thinking in the literature. This is because critical thinking is a multidisciplinary concept that encompasses philosophical, cognitive, and psychological perspectives. It also includes various pedagogical approaches. Philosophically, critical thinking is defined as the principles of good thinking, which involve rationality and intellectual virtues (Paul and Elder, 2013). From a psychological perspective, critical thinking is an active and systematic approach to understanding and evaluating arguments, as well as taking initiative. Defined critical thinking from a pedagogical perspective as the “active conceptualization of knowledge to arrive at an answer or conclusion, as a process of applying, analyzing, synthesizing, and evaluating.” According to Facione et al. (2020), the essence of these explanations is that critical thinking is about proving a point or solving a problem through purposeful thinking, such as interpreting meaning or analyzing a situation.

According to Facione (2020), critical thinking skill is the ability to use logical thinking to learn concepts, make decisions, and solve problems. An individual’s tendency to think critically, rather than their abilities or cognitive skills (Halpern and Dunn, 2021), is what makes them a good thinker. Critical thinking disposition refers to the willingness to exhibit critical thinking skills, which involves being aware of one’s own and others’ thinking approaches, actively using cognitive skills such as reasoning and problem-solving, and desiring to acquire and use new information (Zhang, 2003; Güner and Gökçe, 2021). Critical thinking dispositions are intrinsic motivations to act in accordance with people, events, or circumstances. Individuals with critical thinking dispositions are curious, inquisitive, prudent, truth-seeking, self-confident, open-minded, analytical, and systematic thinkers (Facione et al., 2000). Ennis (2020) similarly defines critical thinking dispositions as: Beliefs and decisions should be based on objective evaluations and a realistic understanding of the situation, while valuing all individuals. Critical thinking disposition is a process that enables people to make informed decisions about their beliefs and actions, and is essential for developing critical thinking skills (Facione, 2020). According to researchers, some individuals possess critical thinking skills but fail to utilize them (Yılmaz, 2021). Accordingly, individuals should possess both critical thinking tendencies and skills (Kezer et al., 2016).

Critical thinking dispositions are characterized by open-mindedness, flexibility, objectivity, and a willingness to research and evaluate different perspectives (Yeşildağ Hasançebi, 2021). It is crucial for individuals to be able to adapt to events and situations, generate alternative solutions, and consider them from various perspectives (Hooks, 2010; Söylemez, 2016; Altın and Saracaloğlu, 2018). To solve problems effectively, it is important to have knowledge of various solution strategies, the ability to switch between them as needed, and the skill to identify effective alternative solutions (Xu et al., 2014).

During the university years, individuals undergo a formative period that shapes their lives, preferences, and decisions. Academic and social relations are intense, and students may encounter various challenges, from adapting to a new environment and system to managing interpersonal relationships. It is crucial to employ effective strategies and maintain a positive attitude toward alternative solutions when facing these challenges (Güvenç, 2019). University students may possess critical thinking skills and high cognitive flexibility levels, enabling them to bring alternative solutions to problems and adapt to change easily (Serpin Eşiyok, 2016). Conducting this study with university students can provide valuable insights for researchers and stakeholders in higher education.

When examining the literature on cognitive flexibility and critical thinking dispositions, studies were found that associated both concepts with different variables. Specifically, cognitive flexibility was found to be associated with self-efficacy (Kaptanbaş-Gürbüz, 2017), while critical thinking dispositions were associated with happiness (Asıcı and İkiz, 2015), learner autonomy and reflective thinking (Orakcı, 2021), stress levels (Altunkol, 2011), and the tendency to ask for help (Koç and Mehdiyev, 2022). In studies conducted on various topics such as critical thinking dispositions, life satisfaction, cultural intelligence, professional satisfaction, emotional reactivity levels, and lifelong learning tendencies, researchers have found significant results (Yelpaze and Yakar, 2019; Yazgan, 2021; Üzümcü and Müezzin, 2018; Yaşar Ekici and Balcı, 2019; Sert Orhan, 2023). For instance, Demir and Demir (2018) investigated the relationship between critical thinking dispositions and cognitive awareness. The following concepts were examined in this study: cognitive awareness (Demir and Kaya, 2015), executive cognition (Özbey and Şahin, 2018), learning styles (Yeler and Ocak, 2021), social emotional learning (Arslan and Demirtas, 2016). This fragment of text discusses various topics including attitudes toward socioscientific issues (Yılmaz and Salman, 2022), lateral thinking (Yıldız and Yılmaz, 2020), autonomy (Uyar and Güven, 2020), learning styles (Avaroğulları and Şaman, 2020), and creativity (Türkmen, 2014). Demirtaş et al. (2023) investigated the cognitive flexibility and critical thinking tendencies of pre-service religious education teachers. Güner and Gökçe (2021) explored the relationship between cognitive flexibility, mathematics achievement, and anxiety. Meanwhile, Toprak et al. (2024) studied the mediating role of cognitive flexibility and critical thinking in the relationship between academic motivation and fear of negative evaluation. Ereglioglu et al. (2023) they conducted a study on perceived parental behaviors and cognitive flexibility as predictors of critical thinking. No study in the literature has examined the relationship between university students’ cognitive flexibility levels and critical thinking dispositions in terms of variables, nor their predictive power. Larsson (2017) highlighted the unresolved issue of critical thinking in pedagogy. El Soufi and See (2019) noted the lack of explicit and systematic teaching of such skills in undergraduate education. Eskin (2014) identified cognitive flexibility as a crucial life skill encompassing affective, behavioral, and cognitive elements. Therefore, examining the relationship between these two concepts can contribute to the literature.

In this study, it is aimed to examine the critical thinking dispositions and cognitive flexibility of university students and whether they differ according to various variables. In line with this purpose, in this study, university students; (1) what is their level of critical thinking dispositions? (2) Do their critical thinking dispositions differ according to gender, class and faculty? (3) what is their level of cognitive flexibility? (4) Do their cognitive flexibility differ according to gender, grade and faculty? (5) What is the relationship between their critical thinking dispositions and cognitive flexibility? (6) Do their cognitive flexibility predict their critical thinking dispositions? (6) do their cognitive flexibility predict their critical thinking dispositions? The main hypothesis of the study is that there is a significant and positive relationship between university students’ cognitive flexibility and their critical thinking tendencies.

## Materials and methods

This study examines the relationship between university students’ cognitive flexibility and their critical thinking tendencies. The study was conducted based on the relational survey model. Relational research is an analysis technique in which variables and parameters are interrelated and information is systematically integrated as theories begin to develop (Cohen et al., 2007, p. 16). There are three things to look for in every relationship analysis. These are; whether there is a relationship, the meaning of the relationship, the direction of the relationship and the level of the relationship (Karasar, 2018, p. 271). At the same time, statistical techniques such as correlation and regression are used to analyze the relationship between the variables measured in such studies. Based on one variable, the other variable can be predicted (Fraenkel and Wallen, 2006).

### Working group

In the study, maximum diversity sampling, one of the purposeful sampling types, was used to determine the study group. The purpose of choosing this sampling is to reveal different dimensions of the problems or phenomena that may arise and to reflect the diversity of individuals to the maximum extent (Yıldırım and Şimşek, 2013). The population of the study consists of students studying at Mersin University. Accordingly, the study was conducted by reaching 366 students studying at Mersin University in the 2023–2024 academic year. The university students participating in the study are studying at different faculties and grade levels of the university.

The study aims to emphasize the importance of examining the effects of demographic variables such as gender, grade level and faculty. These demographic variables were determined in line with the literature. Research shows that there may be gender differences in critical thinking disposition and cognitive flexibility (Facione, 2020). Gender is a factor that indicates that individuals may develop different intellectual processes in line with social roles and expectations. For example, gender norms may affect the way individuals develop or use critical thinking skills. Therefore, understanding the possible effects of gender on cognitive flexibility and critical thinking dispositions is critical to identify the challenges or advantages that different gender groups face in educational processes. Grade level may have a significant impact on students’ cognitive development and critical thinking skills. In general, as the level of education increases (i.e., from freshman to senior year), students’ critical thinking skills and cognitive flexibility tend to increase (Ennis 2020). Grade level reflects students’ level of academic maturity and experience. Students at different grade levels face different educational challenges and develop different strategies to overcome these challenges. This can help us understand how cognitive flexibility and critical thinking skills are shaped. For example, first grade students may focus on more basic skills, while upper grade students may encounter situations that are more complex and require critical thinking. Examining how these differences affect cognitive flexibility and critical thinking dispositions by grade level is also important for developing educational policies. The faculty variable is directly related to the content and quality of the education students receive. Different faculties may differ in terms of the academic disciplines and ways of thinking that students are exposed to. The faculties to which students belong may also have an impact on critical thinking and cognitive flexibility. For example, students in faculties of social sciences, arts and literature often encounter more courses that promote critical thinking and cognitive flexibility, while students in engineering and science faculties may receive a more analytical and problem-solving oriented education. However, these generalizations may not always be valid due to individual differences and program differences between universities (Facione, 2020; Ennis 2020). It is thought that investigating these differences may contribute to the literature.

Data were collected from Mersin University because it is easily accessible and necessary ethical permissions were obtained. The fact that the study was collected from a single university can be considered as a limitation. For this reason, the data obtained from the study are specific to this region and may not be generalizable to other regions, countries or continents. The necessary permissions were obtained from Mersin University Ethics Committee for the conduct of the study.

The demographic distribution of the study group according to gender, grade and faculty status is presented in Table 1.

**Table 1:** Demographic data of the study group.

Variables		N	%
Gender	Female	285	78.1
	Male	80	21.9
Class	1st grade	240	65.8
	2nd grade	24	6.6
	3rd grade	15	4.1
	4th grade	34	9.3
	Formation (graduate)	52	%14.2
Faculty	Education	68	18.6
	Human and Social Sciences	101	27.7
	Science	24	6.6
	Nursing	37	10.1
	Formation	135	37

Of the university students who participated in the study, 285 (78.1%) were female, 80 (21.9%) were male; 240 (65.8%) were first-year students, 24 (6.6%) were second-year students, 15 (4.1%) were third-year students, 34 (9.3%) were fourth-year students, and 52 (14.2%) were graduate students continuing their formation education at the university. Of the participants, 68 (18.6%) were education students, 101 (27.7%) were humanities and social sciences students, 24 (6.6%) were science students, 37 (10.1%) were nursing students, and 135 (37%) were formation students studying in different faculties.

### Data collection tools

In the study, a data collection tool consisting of two parts, a personal information form containing information about the participants and a scale form in which the participants expressed their opinions, was used. In the personal information form, gender, class and faculty information of the participants were included. The scale form was determined by taking into account the use of scales that reveal the views of the participants in line with the purpose of the study, which have been applied in the literature for a long time and whose validity and reliability have been confirmed in many studies. In this direction, information about the personal information form and scales used in the study are presented below.

### Personal information form

The personal information form was prepared by the researcher by determining the variables in line with the literature by taking the opinions of two field experts, one from the critical thinking course and the other from the field of psychological counseling and guidance. The form includes survey questions prepared to determine the participant’s gender, class and faculty information.

### Cognitive flexibility inventory

The Cognitive Flexibility Inventory (CFI) was developed by Dennis and Vander Wal (2010) to assess the ability of individuals to develop alternative thoughts in the face of difficult situations. Adaptation, validity and reliability studies of the scale to Turkish culture were conducted by Gülüm and Dağ (2012). The scale consists of 20 items and two subscales (alternatives subscale and control subscale). The alternatives sub-dimension measures the ability to perceive situations that arise throughout life and possible alternatives to human behaviors and the ability to produce many solutions to solve difficult situations. The control subscale measures the tendency to perceive the ability to control difficult situations. The scale was prepared in a five-point Likert scale and the items are scored between 1 and 5 points (not at all appropriate, not very appropriate, undecided, appropriate, completely appropriate). The highest score that can be obtained from the scale is 200 and the lowest score is 20. It is accepted that the higher the score, the higher the cognitive flexibility. In the Turkish adaptation of the scale, Cronbach’s alpha value was 0.90, the alternatives sub-dimension was 0.89 and the control sub-dimension was 0.85 (Gülüm and Dağ, 2012). Based on the study group of this research, the alternatives sub-dimension was calculated as 0.88 and the control sub-dimension as 0.86 and 0.89 for the whole scale.

### Critical Thinking Disposition scale

The Critical Thinking Disposition (CTD) scale developed by Semerci (2016) consists of 49 items and five sub-dimensions: metacognition, flexibility, systematicity, perseverance-patience and open-mindedness. The scale is graded on a five-point Likert scale and the items are scored between 1 and 5 points (strongly disagree, mostly disagree, partially agree, mostly agree, completely agree). Cronbach’s Alpha coefficient was calculated according to the sub-dimensions: 0.899 for metacognition, 0.892 for flexibility, 0.903 for systematicity, 0.836 for perseverance-patience, 0.672 for open-mindedness and 0.96 for the whole scale. Based on the study group of this research, it was calculated as 0.88 in metacognition, 0.87 in flexibility, 0.89 in systematicity, 0.84 in perseverance-patience, 0.66 in open-mindedness and 0.96 for the whole scale.

### Data collection process

Before data collection, all students involved in the study were informed with a comprehensive explanation of the purpose of the research. Furthermore, participants were assured that the research would have no physical or psychological impact. Furthermore, it was specifically stated that the results of the research would not affect school grades and that students’ names would not be collected. Data collection tools were collected online from the students via Google Form in a classroom setting. Participation in the study was voluntary. It took an average of 15 min to complete the scales.

### Data analysis

SPSS 25 program was used for data analysis. Missing data were checked before the analysis and no missing data were found. In logistic regression, outliers were checked for independent variables. In the independent variables, z scores for univariate outliers and Mahalanobis distance values for multivariate outliers were calculated. While z standard values outside the range of −3.29 to +3.29 are expressed as univariate outliers, Mahalanobis distance values with a probability less than *p* = 0.0001 are expressed as multivariate outliers (Tabachnick and Fidell, 2007). As a result, when the z standard values and Mahalanobis distance values were analyzed, 1 participant was identified above the critical value in the chi-square table and excluded from the study. Another condition for logistic regression analysis is the absence of multicollinearity between variables. If the correlation between variables (*r* > 0.90) is high, the problem of multicollinearity arises and it is accepted that there is a multicollinearity problem between independent variables when the tolerance value is less than 0.10 and the VIF value is greater than 10 (Tabachnick and Fidell, 2007). The data of this study were analyzed in this context and tolerance values were found to be greater than 0.10 and VIF values were found to be less than 10. Therefore, it was determined that there was no multicollinearity problem among the independent variables. Skewness and kurtosis coefficients were calculated to determine the conformity of the remaining 365 data to normal distribution. Since the calculated kurtosis and kurtosis coefficients were in the range of −1.5 and + 1.5, it was accepted that the data were normally distributed (Tabachnick and Fidell, 2007). The results of the assumption tests are shown in Table 2.

**Table 2:** Results of assumption tests.

Analysis step	Test applied	Criteria	Result
Missing data check	–	–	No missing data were found.
Outlier check for ındependent variables	Z score (univariate outliers)	−3.29 < z < 3.29	1 participant identified as an outlier and excluded from the study.
	Mahalanobis distance (multivariate outliers)	*p* < 0.0001	
Multicollinearity check	Correlation	*r* < 0.90	No multicollinearity problem detected.
	Tolerance value	> 0.10	
	VIF (Variance Inflation Factor)	< 10	
Normality check	Skewness and Kurtosis Coefficients	−1.5 < Skewness, Kurtosis <1.5	Data were considered normally distributed.

The tests and criteria used are based on guidelines provided by Tabachnick and Fidell (2007).

This study investigates the cognitive flexibility and critical thinking skills of university students and aims to determine the extent to which students’ cognitive flexibility and critical thinking skills differ according to variables and the relationships between variables. In this context, independent samples *t*-test was used to compare two independent groups and analysis of variance (ANOVA) was used to compare more than two independent groups. After one-way ANOVA, LSD test was used to determine the significant differences between the two groups when the variances were equal and Games-Howell test was used in the findings that violated homogeneity. Arithmetic mean and standard deviation values were calculated to determine the participants’ perceptions of the variables. Pearson correlation coefficients were calculated to examine the relationships between variables. Regression test was utilized to reveal the predictive relationships between variables. The significance level of the study was taken as 𝛼 = 0.05.

## Results

The level of cognitive flexibility and critical thinking tendencies of university students within the scope of the research, whether they differ according to some variables and the relationship between these two variables and the findings regarding the level of cognitive flexibility in predicting critical thinking disposition were presented.

### Findings on university students’ cognitive flexibility

In the study, mean and standard deviation values of students’ cognitive flexibility levels were analyzed. The findings obtained are presented in Table 3.

**Table 3:** Findings related to students’ cognitive flexibility levels.

Variables	n	Mean	SS
Cognitive flexibility	365	3.80	0.487

In Table 3, the mean cognitive flexibility of the students was (𝑥̅= 3.80) and their cognitive flexibility can be considered relatively high. In the study, independent samples *t*-test was conducted to determine whether students’ cognitive flexibility varied according to their gender. The results of the analysis are presented in Table 4.

**Table 4:** Comparison of cognitive flexibility levels of students according to gender.

Variable	Gender	n	Mean	SS	sd	*t*	*p*
Cognitive flexibility	Female Male	285 80	3.71 3.99	0.476 0.466	363	−4.64	0.000

When Table 4 is examined, students’ cognitive flexibility shows a significant difference in favor of males (*p* < 0.05). The status of students’ cognitive flexibility according to their grades is presented in Table 5.

**Table 5:** Status of students’ cognitive flexibility levels according to grades.

Class	n	Mean	SS
1	240	3.79	0.485
2	24	3.83	0.512
3	15	3.79	0.680
4	34	3.70	0.315
Formation (graduate)	52	3.74	0.524
Total	365	3.77	0.487
TTotal	365	3.77	0.487

The mean cognitive flexibility of the students was highest in the 2nd grade (𝑥̅=3.83) and lowest in the 4th grade (𝑥̅=3.70). The total mean was (𝑥̅=3.77). One-way analysis of variance was performed to determine whether students’ cognitive flexibility varied according to their grades. The results of the analysis are presented in Table 6.

**Table 6:** Comparison of students’ cognitive flexibility levels by grade.

The source of the outcry	Sum of squares	Sd	Mean square	*F*	*p*	Difference
Between groups	0.395	4	0.099	0.413	0.799	-
In-group	86.087	360	0.239			
Total	86.482	364				

There was no statistically significant difference in students’ cognitive flexibility levels according to grades (*F* = 0.413; *p* > 0.05). The status of students’ cognitive flexibility according to faculties is presented in Table 7.

**Table 7:** Status of students’ cognitive flexibility levels according to faculties.

Faculty	n	Mean	SS
Education	68	3.74	0.537
Human and social sciences	101	3.68	0.408
Science	24	3.70	0.380
Nursing	37	3.77	0.631
Formation	135	3.88	0.475
Total	365	3.77	0.487

The faculty with the highest mean of students’ cognitive flexibility is formation (𝑥̅=3.88) and the lowest is human and social sciences (𝑥̅=3.68). The total mean was (𝑥̅=3.77). One-way variance analysis was performed to determine whether students’ cognitive flexibility varied according to faculties. The results of the analysis are presented in Table 8.

**Table 8:** Comparison of cognitive flexibility levels of students according to faculties.

The source of the outcry	Sum of squares	Sd	Mean square	*F*	*p*	Difference
Between groups	2.497	4	0.624	2.675	0.032	5–2
In-group	83.99	360	0.233			
Total	86.482	364				

1. Education, 2. Human and Social Sciences, 3. Science, 4. Nursing, 5. Formation.

It can be said that students’ cognitive flexibility levels differed statistically according to faculties (*F* = 2.675; *p* < 0.05). Games-Howell test was conducted to determine the difference of students’ cognitive flexibility levels according to faculties because the groups were not homogeneous. According to the results of the analysis, it can be said that students studying in the formation group have higher cognitive flexibility than students studying in humanities and social sciences.

### Findings on critical thinking tendencies of university students

In the study, mean and standard deviation values of students’ critical thinking dispositions were analysed. The findings obtained are presented in Table 9.

**Table 9:** Findings related to students’ critical thinking tendencies.

Variables	n	Mean	SS
Critical thinking tendencies	365	4.00	0.470

In Table 9, the mean of students’ critical thinking dispositions was (𝑥̅= 4.00) and this value can be considered relatively high. In the study, *t*-test for independent samples was conducted to determine whether the critical thinking dispositions of the students varied according to their gender. The results of the analysis are presented in Table 10.

**Table 10:** Comparison of critical thinking tendencies of students according to gender.

Variable	Gender	n	Mean	SS	sd	*t*	*p*
Critical thinking tendency	Female Male	285 80	4.01 4.00	0.449 0.532	363	0.156	0.876

When Table 10 is analysed, critical thinking dispositions of the students do not show significant difference according to gender (*p* < 0.05). The status of students’ critical thinking dispositions according to their classes is presented in Table 11.

**Table 11:** Students’ critical thinking dispositions according to classes.

Class	n	Mean	SS
1	240	196	23.91
2	24	201	15.01
3	15	195	31.37
4	34	185	17.51
Formation (graduate)	52	197	20.31
Total	365	196	22.94

The highest mean of students’ critical thinking dispositions was in the 2nd grade (𝑥̅=201) and the lowest in the 4th grade (𝑥̅=185). The total mean was (𝑥̅=196). One-way analysis of variance was performed to determine whether students’ critical thinking dispositions varied according to their grades. The results of the analysis are presented in Table 12.

**Table 12:** Comparison of cognitive flexibility levels of students according to grades.

The source of the outcry	Sum of squares	Sd	Mean square	*F*	*p*	Difference
Between groups	4686.933	4	1171.733	2.256	0.063	–
In-group	187015.242	360	519.487			
Total	191702.175	364				

No statistically significant difference was found in the critical thinking disposition levels of the students according to the grades (*F* = 2.256; *p* > 0.05). The status of students’ critical thinking dispositions according to faculties is presented in Table 13.

**Table 13:** Students’ critical thinking tendencies according to faculties.

Faculty	n	Mean	SS
Education	68	192	24.620
Human and social sciences	101	189	17.451
Science	24	198	19.720
Nursing	37	197	28.355
Formation	135	202	23.078
Total	365	196	22.95

The faculty with the highest mean of students’ critical thinking tendencies is formation (𝑥̅=202) and the lowest is human and social sciences (𝑥̅=189). The total mean was (𝑥̅=196). One-way analysis of variance was performed to determine whether students’ critical thinking tendencies varied according to faculties. The result of the analysis is presented in Table 14.

**Table 14:** Comparison of students’ critical thinking tendencies according to faculties.

The source of the outcry	Sum of squares	Sd	Mean square	*F*	*p*	Difference
Between groups	11369.954	4	2842.489	5.675	0.000	5–1 5–2
In-group	180332.221	360	500.923			
Total	191702.175	364				

1. Education, 2. Human and Social Sciences, 3. Science, 4. Nursing, 5. Formation.

It can be said that the critical thinking disposition levels of the students differed statistically according to the faculties (*F* = 5.675; *p* < 0.05). Games-Howell test was conducted to determine the difference of students’ critical thinking disposition levels according to faculties because the groups were not homogeneous. According to the results of the analysis, it can be said that students studying in the formation group have higher critical thinking tendencies than students studying in education and humanities and social sciences.

### Findings on the relationship between cognitive flexibility and critical thinking dispositions

In the study, correlation analysis was performed to determine whether there was a significant relationship between students’ cognitive flexibility and critical thinking dispositions. The data obtained as a result of the analysis are presented in Table 15.

**Table 15:** The relationship between cognitive flexibility and critical thinking dispositions.

Variables	n	*r*	*p*
Cognitive flexibility	365	0.635	0.000
Critical thinking tendency			

There is a significant positive relationship between students’ cognitive flexibility and their critical thinking tendencies (*r* = 0.635, *p* < 0.05). With this finding, the main hypothesis of the study is confirmed.

### Findings on the power of cognitive flexibility to predict critical thinking disposition

In the study, simple regression analysis was performed to determine the degree to which students’ cognitive flexibility predicted their critical thinking dispositions. The findings obtained are presented in Table 16.

**Table 16:** Simple regression analysis results regarding the predictive power of students’ cognitive flexibility for critical thinking dispositions.

Independent variable	Dependent variable	B	R	R2	SE	β	*t*	*p*
Cognitive flexibility	Critical thinking tendency	1.494	0.635	0.405	7.258	0.635	15.648	0.000

Simple linear regression analysis was performed to determine the extent to which students’ cognitive flexibility predicted their critical thinking dispositions. According to the analysis, a significant relationship was observed between cognitive flexibility and critical thinking dispositions (R = 0.635; R2 = 0.405) and cognitive flexibility was found to be a significant predictor of critical thinking dispositions [*F*(1–363) = 244.846, *p* < 0.01]. According to these findings, cognitive flexibility positively and significantly predicts critical thinking disposition and explains 40% of critical thinking disposition.

### General findings on students’ cognitive flexibility and critical thinking tendencies

Information summarizing all the findings obtained from the data obtained from the students is shown in Table 17.

**Table 17:** Students’ cognitive flexibility and critical thinking dispositions.

Factor	Cognitive flexibility	Critical thinking dispositions
Gender	In favor of men	No significant difference
Class level	No significant difference	No significant difference
Faculty	In favor of formation	In favor of formation
Cognitive flexibility level	High	–
Critical thinking disposition level	–	High
Relationship	There is a meaningful relationship (*r* = 0.635, *p* < 0.05).	
Predictive power	There is a significant prediction [*F*(1–363) = 244.846, *p* < 0.01]. Cognitive flexibility explains 40% of critical thinking disposition.	

According to Table 17, students’ cognitive flexibility is significantly different in favor of male and formation group. However, there is no difference according to the grade level. Critical thinking tendencies create a significant difference in favor of the formation group. However, there is no difference according to gender and class level. The cognitive flexibility and critical thinking tendency levels of the participants are high. A significant relationship was observed between cognitive flexibility and critical thinking dispositions (*R* = 0.635) and cognitive flexibility was found to be a significant predictor of critical thinking dispositions [*F*(1–363) = 244.846, *p* < 0.01]. Cognitive flexibility positively and significantly predicts critical thinking disposition and explains 40% of critical thinking disposition.

## Discussion

The cognitive flexibility of university students is relatively high. There are similar studies reaching this conclusion in the literature (Kazu and Pullu, 2023; Pepe, 2021; Yazgan, 2021; Toraman et al., 2020; Kaptanbaş Gürbüz and Sezgin Nartgün, 2018; Camcı Erdoğan, 2018). Since cognitive flexibility is considered as the ability to adapt to situations, the ability to switch between thoughts, or the ability to approach different problems with versatile strategies (Stevens, 2009), this result is important in terms of maintaining students’ communication and social skills in a healthier way and solving problems effectively (Martin et al., 2003).

Students’ cognitive flexibility showed a significant difference in favor of males. There are similar studies in the literature (Sert Orhan, 2023; Koç and Mehdiyev, 2022; Kolburan et al., 2019; Yelpaze and Yakar, 2019; Asıcı and İkiz, 2015; Altunkol, 2011). The result of this study can be explained in terms of socio-cultural context and gender roles because the roles that society attributes to men and women can affect people’s lives. As a matter of fact, in some male-dominated or patriarchal societies, boys are raised by supporting them to deal with problems in a self-confident manner, to find different or contradictory solutions to problems, and to act assertively or aggressively if necessary. Unlike the results of this study, some studies have found that cognitive flexibility levels do not differ by gender (Üzümcü and Müezzin, 2018; Camcı Erdoğan, 2018; Doğan-Laçin and Yalçın, 2018; Kaptanbaş-Gürbüz, 2017; Plukaard et al., 2015; Zahal, 2014).

No statistically significant difference was found in students’ cognitive flexibility levels according to grades. There are studies reaching similar results in the literature (Koç and Mehdiyev, 2022; Yaşar Ekici and Balcı, 2019; Camcı Erdoğan, 2018). However, Başpınar (2019) obtained a different result from the study by concluding that the cognitive flexibility levels of prospective classroom teachers differed significantly according to the grade level.

It can be said that students’ cognitive flexibility levels differ statistically according to faculties. It can be said that formation students have higher cognitive flexibility than students studying in humanities and social sciences. Sert Orhan (2023) obtained similar results with this study by concluding that individuals receiving pedagogical formation education have higher cognitive flexibility levels than students in the faculty of education. The fact that students receiving formation education study in different departments and study in different departments and classroom environments with an interdisciplinary perspective may positively affect their adaptation skills and cognitive flexibility. Yelpaze and Yakar (2019) concluded that the cognitive flexibility levels of the students of the Faculty of Economics and Administrative Sciences and the Faculty of Science and Letters were higher than the students of the Faculty of Education.

Students’ critical thinking tendencies can be considered relatively high. Demirtaş et al. (2023), Akbulut (2019), Bayraktar (2019), Duğan and Aydın (2018), and Akdan (2016) also reached similar results with this study. There are also studies that reached different results. Durnacı and Ültay (2020), Uyar and Güven (2020), Arslan and Ancın (2016), and Can and Kaymakçı (2015) concluded that students’ critical thinking tendencies are not at the desired level. As a matter of fact, Çansoy et al. (2018) examined the academic studies on critical thinking dispositions in Turkey through content analysis. As a result of the studies: It was concluded that students’ critical thinking dispositions are generally low. In this context, in order to eliminate this difference in the literature, it may contribute to the literature to examine students’ critical thinking dispositions in a more in-depth, detailed and different perspective.

Critical thinking tendencies of students do not differ significantly according to gender and grade level. Yıldırım and Şensoy (2017), Avaroğulları and Şaman (2020) and Demirtaş and Kuş (2019) also reached similar results. Unlike this study, Özgün (2019), Türkmen (2014), and Beşoluk and Önder (2010) found a significant difference in favor of girls on critical thinking disposition. Durnacı and Ültay (2020) concluded in their study that gender creates a significant difference in favor of boys in critical thinking disposition. Yıldız and Yılmaz (2020) obtained different results from this study by concluding that critical thinking disposition showed a significant difference according to gender and grade level. It is seen that there are different results in the literature about whether gender makes any difference on critical thinking disposition. Therefore, more specific studies can be conducted in the literature based on the gender variable.

Akkaya et al. (2018) and Karaman (2016) obtained similar results with this study by concluding that grade level did not make a significant difference on students’ critical thinking disposition. Karalı (2012), on the other hand, obtained different results from this study by concluding that the critical thinking disposition of pre-service teachers showed a significant difference according to the grade level and that the skill and value scores increased significantly as the grade level increased.

It can be said that students’ critical thinking disposition levels differ statistically according to faculties. It can be said that formation students have higher critical thinking tendencies than students studying in education and humanities and social sciences. It is seen that formation students have significant differences in both cognitive flexibility and critical thinking tendency. This may be due to the fact that students who graduated from different faculties were supported academically and pedagogically by the faculties of education in addition to their own fields of science in order to become teachers, and that they made extra efforts to increase their personal and professional development and their high motivation to learn in this direction may have contributed to the development of many skills along with their cognitive flexibility and critical thinking tendencies.

A significant positive relationship was found between students’ cognitive flexibility and their critical thinking dispositions above the medium level. Cognitive flexibility was found to be a significant predictor of critical thinking dispositions. Cognitive flexibility positively and significantly predicts critical thinking disposition and explains 40% of critical thinking disposition. There are studies reaching similar results in the literature. Demirtaş et al. (2023) and Güner and Gökçe (2021) concluded that there is a positive and significant relationship between university students’ cognitive flexibility levels and their critical thinking tendencies. Baysal Doğruluk (2021) also concluded that there is a positive, moderately significant relationship between university students’ cognitive flexibility and metacognitive awareness and critical thinking tendencies. Çuhadaroğlu (2013), on the other hand, considered critical thinking as a predictor variable in his study to determine the cognitive variables predicting cognitive flexibility. However, unlike the result of this study, he concluded that critical thinking was not a predictor of cognitive flexibility. However, in the same study, it was also stated that there are common cognitive characteristics between critical thinking and cognitive flexibility. Martins and Gonçalves (2022) stated that cognitive flexibility directly affects the development of competencies such as reasoning, decision making and problem solving. It can be said that some of the characteristics mentioned here also reflect critical thinking characteristics. As a matter of fact, Söylemez (2016) stated in his study that one of the building blocks of critical thinking is flexibility. However, flexibility is accepted as a dimension of critical thinking (Semerci, 2016). Individuals who tend to think critically can use flexibility as the ability to be open to different knowledge, thoughts and opinions in the process of obtaining information about a problem situation and to apply alternative solutions in different fields in the application of the information obtained (Yüksel et al., 2021). It is seen that individuals who think critically, rather than taking the facts as they are, assimilate them in a way they attribute to themselves by filtering them in line with their own experiences or cognitive predictions, and while doing so, they also develop a special attitude toward the phenomenon in question. In order for cognitive flexibility skills to be fully utilized, an individual needs to filter information through critical thinking. In this case, we can consider critical thinking as a necessary skill when using cognitive flexibility skills, but we can also consider critical thinking as a basic tendency in terms of cognitive flexibility (Çuhadaroğlu, 2013).

It can be said that individuals with critical thinking tendencies have cognitive flexibility and individuals with cognitive flexibility tend to think critically (Demirtaş et al., 2023). In this context, it can be said that cognitive flexibility and critical thinking characteristics are related to each other. Based on the results of the study, cognitive flexibility and critical thinking tendencies can be examined more specifically by limiting them in terms of faculty and grade level. It is thought that examining these two concepts with qualitative methods such as phenomenology and case studies can contribute to the literature in terms of providing a broader, deeper, richer and multiple perspectives.

In order to increase the generalizability of the study, research can be conducted on larger and more diverse sample groups from different universities and geographical regions. Longitudinal studies can be conducted to examine the change in cognitive flexibility and critical thinking dispositions over time. Such studies would allow us to better understand how these skills develop throughout students’ academic careers and the effects of demographic variables on this process. Educational interventions or programs can be designed to develop cognitive flexibility and critical thinking skills, and the effects of these interventions can be tested through experimental studies. The effects of such interventions on different demographic groups can also be examined. To gain a deeper understanding of the impact of the faculty variable, the cognitive flexibility and critical thinking dispositions of students in different academic disciplines can be compared. These comparisons may reveal the role of different disciplines in the development of these skills.

Educational institutions can design inclusive education programs to develop cognitive flexibility and critical thinking skills. These programs can aim to provide equal opportunities to all students, taking into account their gender, grade level and faculty differences. Faculty-specific educational strategies can be developed to improve the cognitive flexibility and critical thinking skills of students from different faculties. For example, problem-solving approaches can be encouraged for students in engineering faculties, while critical discussions can be encouraged for students in social sciences faculties. Universities can offer guidance and counseling services for students who want to develop cognitive flexibility and critical thinking skills. These services can be customized according to the individual needs of students. Instructors could be further trained on how to support students’ cognitive flexibility and critical thinking skills. This could encourage instructors to incorporate strategies to develop these skills in their lesson plans and teaching methods.

## Data Availability

The raw data supporting the conclusions of this article will be made available by the authors, without undue reservation.
